# Immodest Dress

**Published:** 1914-04

**Authors:** 


### Immodest Dress

A good many of our contemporaries, lay and medical, are incriminating the modern fashions of dress as tempting to immorality. In particular, respectable women are denounced for making displays which a modest man would consider abnormal except among the demi-monde. Much the same diatribes have appeared in the past, about other fashions, new in their time, and we need not consider whether we can remember these diatribes personally or whether we learn of them from history, more or less ancient. At first thought, we are inclined to agree with these denunciations. But, on more critical consideration, we are impressed with the inconsistency that fashions that revealed have been subjected to equally scathing remarks with those that concealed. The Mother Hubbard gown and the bustle, for instance, have been as strongly denounced on the ground of immodesty as tight-fitting garments. A cynical remark, which may have been the work of a professional humorist seeking the two-dollar fee for a joke, or which may have been genuine, is worth considering. A girl, asked as to whether she did not consider her dress less modest than her mother's at the same time of life, replied in the negative, saying that the modern girl associated less indelicacy with her knees than the one of the past generation did with her ankles. This reminded us that an old lady had once said that, when she was a girl, it was considered immodest to display the ears—an explanation of the curious hair dressing seen in daguerreotypes that was new to us. It certainly is an advance toward true morality to eliminate any sense of sexuality from the ears and, if we consider the matter impartially, there is no particular reason why such aggregations of bone, muscle, etc., as the legs should be concealed by either sex, or why they should not be mentioned or even complimented in the same spirit as the arms, neck, or the parts of the face. Of course, it may be argued that we must draw the line somewhere, but a comparative study of customs in dress shows no special connection between concealment and morality, unless it be to support the contention that the nearer we approach to nakedness the more moral a race becomes.

